# How Do You Say ‘Hello’? Personality Impressions from Brief Novel Voices

**DOI:** 10.1371/journal.pone.0090779

**Published:** 2014-03-12

**Authors:** Phil McAleer, Alexander Todorov, Pascal Belin

**Affiliations:** 1 School of Psychology, College of Science and Engineering, University of Glasgow, Glasgow, United Kingdom; 2 Department of Psychology, Princeton University, Princeton, New Jersey, United States of America; 3 Voice Neurocognition Laboratory, Institute of Neuroscience and Psychology, College of Medical, Veterinary and Life Sciences, University of Glasgow, Glasgow, United Kingdom; 4 Département de Psychologie, Université de Montréal, Montréal, Quebec, Canada; 5 Institut des Neurosciences de La Timone, Université Aix-Marseille, Marseille, France; Northwestern University, United States of America

## Abstract

On hearing a novel voice, listeners readily form personality impressions of that speaker. Accurate or not, these impressions are known to affect subsequent interactions; yet the underlying psychological and acoustical bases remain poorly understood. Furthermore, hitherto studies have focussed on extended speech as opposed to analysing the instantaneous impressions we obtain from first experience. In this paper, through a mass online rating experiment, 320 participants rated 64 sub-second vocal utterances of the word ‘hello’ on one of 10 personality traits. We show that: (1) personality judgements of brief utterances from unfamiliar speakers are consistent across listeners; (2) a two-dimensional ‘social voice space’ with axes mapping Valence (Trust, Likeability) and Dominance, each driven by differing combinations of vocal acoustics, adequately summarises ratings in both male and female voices; and (3) a positive combination of Valence and Dominance results in increased perceived male vocal Attractiveness, whereas perceived female vocal Attractiveness is largely controlled by increasing Valence. Results are discussed in relation to the rapid evaluation of personality and, in turn, the intent of others, as being driven by survival mechanisms via approach or avoidance behaviours. These findings provide empirical bases for predicting personality impressions from acoustical analyses of short utterances and for generating desired personality impressions in artificial voices.

## Introduction

Voices are saturated with cues to a person's age, gender, and affective state [1], with information being extractable whether listening to sentences [2], or sub-second vocal bursts [3], [4]. Within voice perception, a focus on personality has endured: from Cicero's apparent pondering of competent speakers in *De Oratore*; through the golden period of radio exploring status [5]; to modern researchers examining various personality traits including attractiveness and dominance [6]–[12].

Judgements of personality influence our social interactions. By example, perceived facial attractiveness affects numerous decisions that we make (for review see [13]), including mate choices, job selection and voting behavior [12], [14], [15]. Likewise, research has shown that perceived vocal personality influences mate selection, leader election, and consumer choices [16]–[19]. Such judgements from faces are formed after less than 100 ms exposure, [20], [21] and are consistent across observers [22], [23]. Furthermore, given that many judgements are based on static images or short interactions, these decisions are largely made without much knowledge of the person in question – often termed ‘zero acquaintance’ [23]–[27]. Yet, despite their equal relevance to our daily lives, the rapid attribution of personality traits to novel speakers is poorly understood. As such, the key traits for deriving first impressions of people from short vocalizations, and the vocal acoustics governing these traits, remain to be established.

Across various domains, it has been shown that consideration of numerous personality traits may be reduced to summary dimensions, in turn allowing for the estimation of other traits [28]–[30]. Fiske, Cuddy and Glick [31] revealed that judgements of social groups were summarised via a two-dimensional space comprising of warmth and competence. Likewise, Oosterhof & Todorov [32] showed personality impressions from faces were summarized by valence and dominance: Sutherland and colleagues [33] validated this model for faces, whilst also proposing a third dimension of attractiveness-youth. In voices, from scrambled mock-jury deliberations, female judgements of male speakers were summarised by ratings of friendliness and dominance [10], whilst Zuckerman and colleagues [12], utilising people reading passages of texts, found the three key dimensions explaining personality traits to be dominance, likeability and achievement. Furthermore, Montepare & Zebrowitz-McArthur [29] found comparable results exploring personality attribution of people reciting the alphabet. Thus one proposed understanding is that, typically, a two dimensional space can summarise all other traits, with one trait emphasising warmth/trust/likeability, and a second trait emphasising strength/dominance.

Such a solution is clearly influenced by the traits examined. For example, as perhaps a compromise to the numerous possible personality traits [34], and thus overlooking a summary space, many studies of face and voice perception have utilised traits from the Big Five Personality Model [35], [36]. As in studies exploring traits such as trust, intelligence and attractiveness, studies using the Big Five have again shown large consistency between viewers' ratings, as well as accuracy when compared to self-reports e.g. [10], [27], [34], [37]–[41]. Taken together, however, it is evident that humans make use of rapid judgements on connected traits to help guide our interactions [32], [34], [42].

Yet, the purpose of evaluative ‘spaces’ extends beyond personality judgements, with a putative role being for the establishment of the intent of others, and in turn, for the triggering of approach/avoidance behaviours by ourselves [32], [43]. This proposition lies in a series of hypotheses based on the overgeneralisations of age, attraction, emotion and familiarity [23], [43]–[45]. Secord [46] proposed that via a temporal extraction of momentary characteristics (such as a smile, or a deep voice) we label people with an enduring attribute, such as friendliness or strength. These generalisations allow for rapid – though not necessarily accurate – judgements of personality in an enriched world and, in turn, for appropriate action in terms of approach/avoidance to be taken. Thus, a judgement on the warmth dimension would evaluate a novel person as a friend or foe, whilst a judgement on dominance dimension would evaluate that person's ability to act on their intent. A generalization from a snapshot image to an enduring attribute appears to hold true for first impressions from faces [23], [32], [47]–[49], and indirectly in voices, using extended speech [6], [11], [12], [29].

However, previous vocal studies differ in comparison from other modalities in terms of the quantity, quality and relevance of the presented signal. Thus far, studies of personality traits of novel speakers have used long ‘irrelevant’ passages of speech (>10 s duration) [12], [29] but see [10], introducing influence from uncontrolled parameters of speech prosody. Studies that do utilise brief and socially relevant stimuli have a sole focus on attractiveness of the speaker, neglecting other potentially important traits [50], [51]. In contrast, face perception emphasises a ‘first impression’ scenario via rapid presentation of static faces (<100 ms duration). Thus it is pertinent to establish if a two-dimensional space holds true for short, socially relevant, vocal signals from novel speakers, akin to a ‘first impression’. From there, it would be possible to establish the acoustical properties of such judgements and perceived personalities. By extrapolation thus, if a brief vocal signal (sub 1 second) is akin to a static face [1], then given reported similarities in voice processing [1], [52] and face processing [53], one may propose that a two-dimensional space would explain first impression judgments of personality from voices.

This paper investigates the personality traits conveyed by novel speakers, via a single word, in an ambiguous scenario. We tested whether personality ratings, for both male and female voices, would be consistent across listeners, and if so, would they be appropriately summarized by a two-dimensional ‘social voice space’, similar to previous findings in face perception. Furthermore, given the lack of understanding of the underlying acoustics of such spaces, eight acoustical measures, summarising voice production, were tested for a relationship to any resultant summary spaces.

## Methods

### Ethics statement

All procedures (recording and experimental) were approved by the University of Glasgow ethics committee, and it was conducted in accordance with the ethical standards laid down in the 1964 Declaration of Helsinki.

As the experiment was carried out online, participants gave informed consent prior, via first reading a series of statements regarding anonymity, freedom to withdraw, and secured storage of data, and by then clicking an online button to confirm that they have read and agreed to these statements. Participants were not permitted to take part without providing consent. This procedure was approved by the ethics committee of the University of Glasgow.

### Participants

64 speakers (all Scottish; 28.2±10.2 years; 32 male) from the University of Glasgow undergraduate population were selected for stimuli recording. All speakers reported normal hearing and were given a monetary reward or partial course credit. Selection criteria included only people born and raised in Scotland to stabilise any potential effect of speaker provenance.

320 new participants (117 male; 28.5±10.6 years) from the same pool as above took part in the main voice rating experiment. Again all participants were given a monetary reward or partial course credit for taking part.

### Stimuli

All 64 speakers were, individually, digitally recorded (16 bit mono, 44100 Hz, WAV format) reading an unfamiliar passage of text in a soundproof booth. Speakers were instructed to read the passage, involving a telephone conversation with direct speech, in a neutral tone. The word ‘hello’ was extracted from each recording, and normalised for power (RMS) and loudness via Matlab (the Mathworks). Stimuli had an average duration of 391 ms ± 65.1 ms and 390 ms ± 64.1 ms for male and female voices, respectively. ‘Hello’ was selected because it is a familiar, social word, with a medium-to-high range of common usage (British National Corpus) and its position and punctuation allowed for extraction. Cultural equivalents of ‘hello’ have previously been used to study ratings of attractiveness across culture (‘hujambo’ – Swahili, [50]) and across temporal modulation (‘bonjour’ - French, [51]). Example stimuli can be heard at http://vnl.psy.gla.ac.uk/socialvoices.php

### Procedure

The experiment took place online. Participants were recruited via email and directed to a web address. Though no control was established over listening environment, participants were instructed to carry out the experiment in a quiet room using either headphones or speakers attached to the computer. Furthermore, recent research exists that shows data from online experiments is comparable to data from lab-based experiments [54], [55].

Each participant was pseudo-randomly assigned to one of ten rating scales taken from previous literature examining social traits in face, voice and person perception [12], [28], [29], [31], [32]: Aggressiveness, Attractiveness, Competence, Confidence, Dominance, Femininity, Likeability, Masculinity, Trustworthiness and Warmth. Each participant rated only one trait, as opposed to numerous traits e.g., [12], [56], to remove the influence of any halo effects (e.g. rating a speaker high on warmth would in turn make it difficult to rate that voice low on likeability).

For each stimulus, participants were asked, “Based on the voice, please rate how {TRAIT} is this person” on a 9-point Likert scale, ranging from 1 (extremely un{TRAIT}) to 9 (extremely {TRAIT}). No contextual grounding or scenario for the experiment was given: participants were not informed that the ‘hello’ stimuli they would hear came from longer extracts. After the experiment, participants confirmed they did not recognise any of the voices. Stimuli were blocked by gender and counterbalanced across subjects. Within gender, each voice was heard twice across two discrete blocks – no breaks. All voices were heard once per discrete block with presentation order randomised in both blocks. An untimed break was given prior to the change in gender. The uncompressed sounds were played through a FLASH (www.adobe.com) object interface running on all common open-source web browsers.

### Data analysis

Exclusion criteria, stipulated prior to commencing the study, compensated for the lack of information on subject behaviour during the experiment: 1) that in each subject, two-thirds of the ratings given to the repetitions of each stimulus should fall within two rating points of each other (i.e. a voice rated 5 on first hearing would be later rated between 3 and 7); 2) that no subject should respond the same rating to greater than 75% of all voices (e.g. all voices rated 5). For the ratings of Masculinity and Femininity, criterion 2 was relaxed to 50%. Using these criteria, the data of 10 subjects (3.1%) were excluded.

Data collection occurred over a period of approximately one month. The number of participants per rating scale varied due to: 1) subjects removal; and 2) a technical constraint of the online programme where two subjects commencing at the same time would be assigned to the same trait. Inter-rater reliability is summarised in Table 1: all Cronbach Alphas > 0.88 and inter-rater agreement was considered high for each personality trait assessed.

**Table 1:** Cronbach alpha scores, indicating reliability of judgments, and number of participants per trait judgment.

Social Trait	Cronbach Alpha	N
Aggressiveness	0.98	33
Attractiveness	0.91	31
Competence	0.90	36
Confidence	0.88	34
Dominance	0.88	28
Femininity	0.98	24
Likeability	0.88	30
Masculinity	0.98	25
Trustworthiness	0.93	28
Warmth	0.92	33
Average	0.92	30.2

Alpha greater than 0.85 is considered to be high.

Principal Component Analysis (PCA) was used to convert all traits to orthogonal dimensions. Entered into the PCA were the z-transformed, mean ratings for all voices on each scale. Preliminary analysis indicated gender clustering, consistent with biological differences in male and female voices (e.g. higher average pitch in female voices) [57]. Thus, separate gender-driven PCAs were carried out, excluding masculinity and femininity, and only the gender-driven PCAs are reported: masculinity and femininity relationships to the main principal components (PCs) were explored via post-hoc correlational analyses. In addition, analyses comparing personality ratings across male and female raters listening to male and female voices is available in the Supplementary Information (File S1).

### Acoustical measures

Acoustical measures were extracted from the 64 voice stimuli using PRAAT software (V4.2.07; default settings unless stipulated; http://www.praat.org) [58]. 8 measures were selected, in order to constrain multiple comparisons, that reflected differing parts of voice production and perception [59], [60], across the duration of each sound: 1) mean fundamental frequency/pitch (f0) (range: min 75 Hz; max: 600 Hz); 2) changing f0 (maxf0 minus minf0) as an index of intonation [61]; 3) glide, measured as f0-end minus f0-start; 4) formant dispersion, representing filtration of the sound by the vocal tract and related to vocal tract size (measured as the ratio between consecutive formant means, from F1 to F4 [62] using the Burg linear predictive coding algorithm installed in PRAAT [63] – maximum formant frequency was set to 5.5 kHz; window length  =  0.025 s); 5) harmonic-to-noise ratio (HNR) indicating roughness, via the forward cross-correlation method (mean value; time step  =  0.01 s; min pitch  =  75 Hz; periods per window  =  4.5); 6) jitter, a measure of local f0 variations, via Relative Average Perturbation (RAP) measuring the average absolute difference between a period and the average of that period and its two neighbours (shortest period  =  0.0001 s; longest period  =  0.02 s; max. period factor  =  1.3); 7) shimmer, a measure of amplitude variation, via the Amplitude Perturbation Quotient (APQ3) measuring the average absolute difference between a periods amplitude and the average of amplitude of its neighbours, divided by the average (shortest period  =  0.0001 s; longest period  =  0.02 s; max. period factor  =  1.3; max. amplitude factor  =  1.6); 8) alpha ratio, a measure of the source spectral slope [64] using the ratio of mean energy within low (0–1 kHz) vs. high frequencies (1–5 kHz) computed from the long-term average spectrum [65]. All measurements are taken across the duration of each sound (average 390.5 ms) and thus represent global values: this is inclusive of harmonicity measures, representing an indication of signal-to-noise ratio as calculated within PRAAT. Such measures are similar to those previously utilised in studies comparing animal and human vocalisations [66]. Stepwise Regression analysis (criteria: in p< = .05; out p = >.1) was used to establish a relationship between acoustical measures and PCs.

One note is that the acoustical measures selected may be considered imperfect estimates of values obtained using more standard sustained vowel conditions. For each stimulus, the measures are based on mean estimates across the full duration of the word ‘hello’, and although the word is brief, the measures could potentially be affected by time-varying aspects of speech. That said, the same measures were found to show consistent results across sustained vowels and ‘hello’ samples when examining the neural correlates of norm-based coding of voice identity [65], and therefore should be considered as valid for inclusion in this study.

## Results

### Male voices PCA

**Figure 1:**
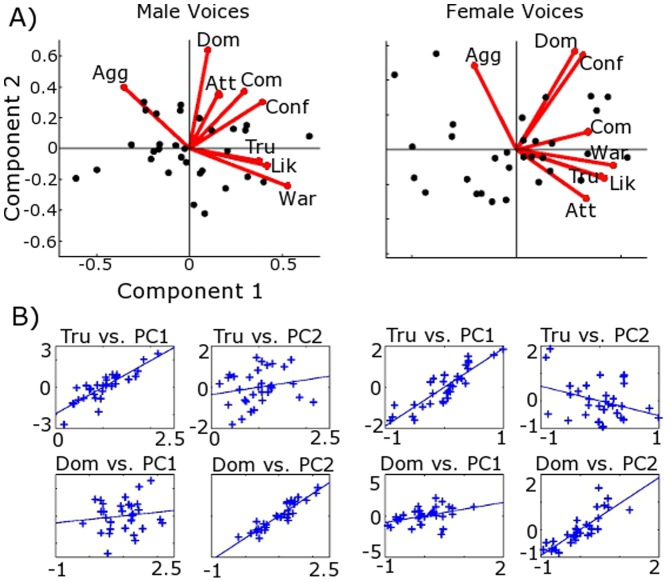
Principal Component Analysis solutions and main correlates of the Social Voice Space. A) The two dimensional solution of the Principal Component Analysis for male (left) and female (right) voices (black dots). Labels equate to: Agg – Aggressiveness; Att – Attractiveness; Com – Competence; Conf – Confidence; Dom – Dominance; Lik – Likeability; Tru – Trustworthiness; War – Warmth. B) Correlation plots between the ratings of trustworthiness (Tru - top row), dominance (Dom - bottom row), and the first (PC1) and second (PC2) principal components for male (left) and female (voices). Blue ‘+’ represent individual voices. Trustworthiness was chosen arbitrarily over Likeability due to the strong correlation between these two traits.

A two-dimensional solution was found to fit ratings for the male voices (without the Femininity and Masculinity ratings), explaining 88% of the variance (56.2% by the first principal component (PC1); 31.8% by PC2; 6.9% by PC3) (see Table 2). All traits, except Aggressiveness, loaded positively with varying strength onto PC1 (see Figure 1a). For PC2, Aggressiveness, Attractiveness, Competence, Confidence and Dominance had positive loadings, whereas Likeability, Trustworthiness and Warmth judgements had negative loadings.

**Table 2:** Loadings on the first two principal components of all social traits for the male and female voice PCAs, including variance explained.

	Male PCA		Female PCA	
Social Trait	Component 1	Component 2	Component 1	Component 2
Aggressiveness	−0.74	0.61	−0.52	0.76
Attractiveness	0.33	0.71	0.74	−0.45
Competence	0.70	0.63	0.88	0.20
Confidence	0.75	0.44	0.62	0.74
Dominance	0.15	0.98	0.55	0.80
Likeability	0.95	−0.20	0.93	−0.24
Trustworthiness	0.92	−0.05	0.96	−0.15
Warmth	0.91	−0.35	0.91	−0.12
Variance Explained (%)	56.18	31.8	59.54	25.53

Loadings represent the correlations of the trait judgements with the first two principal components as calculated including all eight personality traits.

To establish summaries of the principal components, repeated PCAs were performed systematically removing individual scales as likely candidates, and correlating the new PCs to the removed personality scales. An original scale is proposed as a suitable summary if it correlates strongly with one PC and weakly with the other. PC1 of all ratings excluding Trustworthiness, highly correlated with Trustworthiness ratings (r_s_ = .92, p<.001; Trustworthiness to PC2, r_s_ = −.19, n.s). Likewise, PC1 of all ratings excluding Likeability, highly correlated with Likeability ratings (r_s_ = .95, p<.001; Likeability to PC2, r_s_ = −.3, n.s.). In turn, ratings of Trustworthiness and Likeability were strongly correlated (r_s_ = .93, p<.001). Excluding Dominance, PC2 correlated strongly with Dominance ratings (r_s_ = .94, p<.001; Dominance to PC1, r_s_ = .06, n.s.) (Fig. 1b). A three dimensional solution to this PCA, and analysis based on gender of rater, is shown in the Supplementary Information (File S1; see Table S1 for 3D PCA, and Table S2, Table S3 & Table S4 for analysis by rater gender).

Exploring Masculinity and Femininity ratings to male voices, the all-traits PC1 was positively correlated to Femininity (r_s_ = .63, p<.001) and negatively to Masculinity (r_s_ = −.46, p<.05); PC2 was positively correlated to Masculinity (r_s_ = .50, p<.001) and negatively to Femininity (r_s_ = −.4, p<.05).

### Female voices PCA

Following the same criteria, a two dimensional solution was found to explain 88.1% of the variance (PC1: 59.54%; PC2: 28.53%; PC3: 5.2%). All loadings on PC1 were positive except Aggressiveness. On PC2, Aggressiveness, Competence, Confidence and Dominance were all positive (Table 2). PC1 excluding Trustworthiness was highly correlated with Trustworthiness ratings (r_s_ = .93, p<.001; Trustworthiness to PC2, r_s_ = −.05, n.s.). Excluding Likeability, PC1 was highly correlated with Likeability ratings (r_s_ = .92, p<.001; Likeability to PC2, r_s_ = −.04, n.s.). Again, ratings of Trustworthiness and Likeability were highly correlated with one another (r_s_ = .85, p<.001). PC2, excluding Dominance, was highly correlated with Dominance ratings (r_s_ = .84, p<.001; Dominance to PC1, r_s_ = .51, p<.05). Despite having a moderate correlation to PC1, Dominance was selected as an appropriate summary for female PC2 as the next appropriate trait, Aggressiveness, had a similar relationship to PC1 but a weaker relationship to PC2 (Aggression to PC1, r_s_ = .47, p<.05; Aggression to PC2, r_s_ = .78; Aggression to Dominance, r_s_ = .46, p<.05). A three dimensional solution to this PCA, and analysis based on gender of rater, is shown in the Supplementary Information (File S1; see Table S1 for 3D PCA, and Table S2, Table S3 & Table S4 for analysis by rater gender).

Incorporating Masculinity and Femininity to female voices, a relationship was only found for PC1 in that, as PC1 (Trustworthiness) increased, perceived Femininity increased (r_s_ = .7, p<.001) and Masculinity decreased (r_s_ = −.7, p<.001).

### Acoustical measures

Independently by gender, stepwise regression analyses were performed using eight acoustical measures to explain variance in the first two principal components. For PC1 in male voices, a linear combination of f0 (b = 0.48, p<.05) and HNR (b = −0.57, p<.001), explained 49% of the variance, (R = .7, F(2,29) = 14.05, p<.001); in female voices, HNR (b = −0.44, p<.01), glide (b = −0.58, p<.001) and intonation (b = 0.6, p<.001), explained 68% of the variance in PC1 values (R = .82, F(3,28) = 20.12, p<.001). Regarding PC2, in male voices, a combination of alpha (b = −0.25, p = .06), f0 (b = −.037, p<.05), HNR (b = −0.41, p<.05) and formant dispersion (b = −0.29, p<.05), explained 68% of the variance (R = .82, F(4,27) = 14.2, p<.001); for female voices, dispersion (b = −.43, p<.05) and f0 (b = .34, p<.05) explained 27% of the variance (R = .52, F(2,29) = 5.56, p<0.05).

### Secondary analysis of attractiveness

Across gender, subjective inspection of the original PCA solutions were similar, differing largely only in the weighting of Attractiveness. Looking within gender of speaker, for male voices, perceived Attractiveness was significantly more correlated with PC2 (dominance) (r_s_ = .72, p<.001; PC1: r_s_ = .29, n.s.; t_Difference_  =  8.29, p<0.05). In contrast, for female voices, perceived Attractiveness was significantly more correlated with PC1 (valence) (r_s_ = .74, p<.001; PC2: r_s_ = −.45, p<.05; t_Difference_  =  6.35, p<0.01). Across gender of speaker, perceived female vocal attractiveness was significantly more correlated to PC1 than male vocal attractiveness (t_Difference_  =  2.79, p<0.05). Finally, male vocal attractiveness was significantly more correlated to PC2 than female vocal attractiveness (t_Difference_  =  10.18, p<0.01).

Given that attractiveness can also be viewed as a product of personality traits, and is highly prevalent in the literature (e.g. [6]–[9], [12], [50], [56], [67], [68]), we explored the ability to predict Attractiveness ratings based on the ‘social voice space’, separately for male and female voices. In separate PCA analyses, after removing Attractiveness, personality ratings for both male and female voices were summarised by a two-dimensional space explaining 90% of the variance. For male voices, Likeability, Trustworthiness and Warmth were all strongly correlated with PC1 (all r>0.9, p<.001); Dominance correlated strongly with PC2 (r_s_ = .98, p<.001). For female voices, Likeability, Trustworthiness, Warmth and Competence all had strong correlations with PC1 (all r>0.9, p<.001); Aggressiveness (r_s_ = .84, p<.001) and Dominance (r_s_ = .77, p<.001) had good correlations with PC2.

Stepwise regression analysis showed that a linear combination of PC1 (b = 0.4, p<.01) and PC2 (b = 0.7, p<.01), explained 54% of the variance in male Attractiveness ratings (R = .75, F(2,29) = 19.2, p<.001). Both principal components had a positive influence, suggesting as PC1 (Trustworthiness, Likeability) and PC2 (Dominance) increase, so does perceived male vocal Attractiveness, with PC2 (Dominance) having a marginally stronger influence than PC1 (Trustworthiness). Finally, in females, a similar analysis showed that a linear combination of PC1 (b = 0.76, p<.01) & PC2 (b = −0.29, p<.01), explained 66% of the variance in female attractiveness ratings, (R = .81, F(2,29) = 27.65, p<.001). PC1 had a strong positive influence whilst PC2 had a weak negative influence, suggesting that perceived female vocal Attractiveness is largely influenced by increasing PC1 (Trustworthiness, Likeability, Warmth).

## Discussion

The results showed that from brief utterances containing limited information, akin to a first impression, listeners showed high consistency in their ratings of perceived personality. Furthermore, a two-dimensional ‘social voice space’, with a first dimension (PC1) corresponding to perceived likeability and trustworthiness, aligning with ‘valence’ [32], and an orthogonal dimension (PC2) corresponding to perceived dominance, summarized all perceived personality traits in both genders. Despite limited control of experimental listening environment, results are aligned with findings that observers form consistent and reliable impressions from brief exposure to faces [21], [69], [70] and extracts of extended speech [12], [56]. Moreover, agreement on a number of perceived traits, such as warmth, has been shown across cultures for faces [70] and voices [24]. Similarities across personality spaces in voice [10]–[12], [29] and face perception [32] supports the suggestion that the processing of faces and voices, at both the perceptual and neural level, operates via equivalent comparisons of the available information to each modality [1], [52], [53], [71].

The ‘social voice spaces’ witnessed are not only consistent across voice gender, with the exception of attractiveness judgements, but are in agreement with dimensional solutions obtained in various studies exploring: semantic relationships in words [28]; scrambled voice percepts and extended extracts [10], [12], [29]; face perception [32]; and intergroup relationships [31]. These dimensional spaces map strongly with each other when collapsing interchangeable names such as valence and social goodness, or dominance and strength. Each dimensional solution contains an element of positivity or trust, and an element of ability or competence to act. The current use of short socially relevant vocal bursts highlights the validity of these dimensions in establishing first impressions from voices.

Across gender, only the PCA weighting of attractiveness appeared to vary largely. Male vocal attractiveness correlated most strongly with dominance, whilst female vocal attractiveness was most associated with valence. When attractiveness was explored as a product of the traits, i.e. as opposed to an individual trait, components of dominance and valence explained greater than half the variance in male vocal attractiveness: dominance having the stronger influence. In contrast, in female voices, components of valence and dominance/aggression explained almost all of the variance, with the valence component having the strongest effect. These results were largely consistent when exploring the relationship by gender of rater. Previous research has suggested similar results in face [72] and voice perception [68], [73], with findings pointing to increased attractiveness as masculinity/strength increases in males and as friendliness/warmth increases in females.

This study indicates that estimates of attractiveness can occur rapidly, from a brief signal, and the bases of these estimates are consistent with relationships witnessed from hearing longer speech extracts. However, it is worth noting that despite the prevalence of study of vocal attractiveness, it was not one of the two key traits in the PCA, and thus its role is potentially minimal when establishing a first impression of a novel speaker. A three dimensional PCA solution of the current study suggested attractiveness may be related to PC3, though the explained variance was small and any relationship was not significant: in turn, supporting a two dimensional solution. However, attractiveness as a third dimension has been indicated via a validation study of the Oosterhof and Todorov face personality model [32] using 1000 faces [33]. Thus the role of attractiveness should not be marginalised without further study.

Parsing out the true relationship of trustworthiness, dominance and attractiveness, and how we utilise the available signal to make such judgments, may be possible via modern methods of stimulus morphing and averaging [32]. For example, it is known that averaging both faces and voices can increase attractiveness [7], [74], [75]; largely due to smoothing of the respective signal. In turn increased attractiveness can increase trustworthiness though the two are not necessarily directly related [76], [77]. Additionally, at the neural level, it has been shown that we make judgements of identity and attractiveness based on stored prototypes [65], [74], [78]–[80]. For voices, this prototype is explained by at least two of the acoustical variables that partially determine trustworthiness, dominance, and attractiveness - namely f0 and dispersion [65]. Therefore, it is possible that personality perception also relies on comparison to a prototype at least similar, if not the same, as the one used to establish identity. Furthermore, given the consistency of personality ratings across participants, such a prototype would not necessarily be specific to an individual, but may share common properties within a culture.

Analysing the underlying acoustical information, intonation, glide, and HNR were involved in explaining valence in female voices while pitch and HNR explained valence in male voices. For females, a more positive perceived valence appears associated with a greater rise in pitch between the first and second vowel of the word ‘Hello’ (rising intonation); a more negative valence is associated with a falling intonation. The relationship between intonation and valence aligns with a connection reported between facial features and valence, e.g. facial expression [23], [32]: both vocal intonation and facial expression are malleable features of their respective modalities, and these transient adjustable features may drive percepts of valence. For males, an average higher pitch relates to increased valence: this would bring the pitch closer to that of females, resulting in increased friendliness due to stereotyping [81]. The association with HNR in both genders may relate to changes in age: decreasing HNR has been proposed in vocal aging, either chronological or physiological [82], though findings are inconclusive [83]. It is possible that older voices are perceived as more friendly/trustworthy, than younger voices, though this would conflict with reports that younger voices are perceived as warmer, more honest and less dominant [6], [11], [29]. Discrepancies with previous studies may result from the use of longer speech patterns introducing additional parameters known to influence trait impressions, e.g. speech rate [18], [73].

In perceived male vocal dominance, associations were found with decreasing average pitch and formant dispersion, along with decreasing alpha and HNR; decreasing formant dispersion was also associated with female dominance, along with increased average pitch. Thus, lower pitched male voices, across the sound duration, were perceived as more dominant; conversely, higher pitched male voices were perceived as less dominant. In contrast, higher average pitch was associated with increased dominance in female voices. Extensive research shows that listeners are adept at judging various physical characteristics of a speaker from their voice, such as age, height, weight, and body shape, to a varying degree of accuracy [5], [73], [84]–[89]. Such ability may have arisen via adaptation mechanisms in terms of projection of a desired status, culture, or of suitability for mate selection [84]–[86], [90]. The relationship found in male voices in the current study is in-keeping with reports that pitch is often erroneously used to distinguish powerful characteristics such as height, strength and leadership [16], [91]. People assume lower pitch equates to increased strength, particularly in males, due to misconceptions regarding the vocal system structure [91]. The pitch/dominance link may reflect this at a personality level. In reality, formant dispersion is a better gauge as it relates more closely to vocal tract length [62], [84], [92]. Relationships between formant dispersion and dominance have previously been shown in human and non-human mammals [93], [94], and are re-iterated in this study. Increased average pitch in females is normally associated fecundity [50], not dominance, and the relationship found here should be taken with caution as female dominance was the least explained trait, in terms of variance, by the acoustics predictors. Overall, we suggest that such longitudinal changes in vocal acoustics, (e.g. dispersion, HNR), mirror impressions of dominance and physical strength in faces, signalled by ‘static’ aspects of faces (e.g., facial size, inter-ocular distance etc.) [22], [95].

Overall, we form trait impressions as a means to establishing the intent of others, and of selecting appropriate approach and avoidance behaviours. As witnessed, both in the current paper for voices, and in previous papers for faces, these judgements occur rapidly, which is in-keeping with an evolutionary pressure for their existence. A proposal for their creation, largely studied in face perception, revolves around the over-generalization hypotheses [43], [44], whereby we make judgements based on the extrapolation of momentary states to stable dimensions [32], [46]: i.e. a person who smiles (momentary state) is perceived as warm (stable dimension). Such relationships between emotion and personality in voices are as yet only subjective [6], [11], [29]. That said, utilising novel morphing techniques for vocal sounds [3], [4], [96] would make the link between vocal emotion and vocal personality, a tangible and pertinent line of study.

A possible caveat to the present study is that PCA is directed by its input: an untested trait may have greater influence than the proposed dimensions. However, studies utilising free-response data have ultimately reduced to semantically similar dimensions of Valence and Dominance [12], [28], [32]. Thus, in the current work, Valence and Dominance remain strong candidates as the foundations of rapid trait impressions for novel speakers in an ambiguous scenario.

Additionally, the accuracy of first impression judgements remains questionable. Accuracy is an important aspect as if people’s judgements of personality were continually wrong then any subsequent impression of intent based on this perceived personality would be misleading. Typically, accuracy is determined via convergence between self-ratings and ratings by acquaintances. Previously, results have shown only moderate convergence at best, and for a limited number of traits such as dominance and honesty [23], [44], [97]. One problem with trait attribution is the assumption of context-independent personality. People may accurately infer the momentary state of another, but the same inference may not hold when generalised across situations and time. Thus, in order to establish how accurate we are in determining the personality of others, a context-based measure of accuracy would be more appropriate [98].

Finally, the question of consistency of voice personality over time and delivery should be addressed. In the current study we utilised a socially relevant, one word sample of direct speech, read from a passage, whilst previous research has used either long passages or various exerts of people speaking (scrambled or not) e.g. [5], [10], [12], [29], [56], [86], [99]–[103]. How these methodologies compare is an interesting question. Clearly the longer the passage heard and the more natural the phrasing, the more variables are introduced relating to voice quality which may alter the perceived personality [18], [73], [103]. That said, using read exerts of direct speech maintains content across speakers whilst allowing an element of conversation: research has shown that people engage in a naturalistic manner when reading direct speech, as opposed to indirect speech, and that listeners process it in a fashion similar to when having a conversation [104], [105]. Thus, given the consistency of the current findings to previous studies, it could be hypothesised that our initial impressions of personality will persist, irrespective of the manner and duration of what we hear a person say. This would reflect face literature where personality judgements from brief exposures to static faces are consistent to those from longer exposures or from dynamic videos of faces [21], [106]. Taken together, these findings would reiterate the importance of establishing a good first impression.

## Conclusions

Listeners show high agreement when deriving first impressions of novel speakers. A two dimensional ‘social voice space’, constructed via ratings of Valence and Dominance, allows for the extrapolation of all other traits, regardless of gender. Acoustical analysis reveals that Valence is related to pitch variation, whereas Dominance is related to more stable parameters. Furthermore, first impression of vocal attractiveness in male voices relates to perceived strength, whilst in females, vocal attractiveness relates to perceived warmth and trustworthiness.

This study provides an empirical basis for the assessment of personality from voice. In establishing the acoustics that drive certain percepts, people and algorithms may be instructed on the necessary alterations to obtain a desired projection: this has endless application in fields as diverse as business, computing, engineering and advertising. Focus must now turn to stability across longer utterances and differing contexts to fully capitalise on the relevance for modernised voice activated/controlled systems, and for understanding how we are influenced by the signals received from others.

## Supporting Information

File S1 **Supplementary Information, Analysis and Interpretation of PCAs.**
(DOCX)

Table S1 **A three dimensional solution to the ‘social voice’ space.**
(DOCX)

Table S2 **Proportion of each gender per personality scale.**
(DOCX)

Table S3 **A three dimensional solution for female voices by rater gender.**
(DOCX)

Table S4 **A three dimensional solution for male voices by rater gender.**
(DOCX)
