# Supplementary material for: How Do You Say ‘Hello’? Personality Impressions from Brief Novel Voices
Source: PLoS One. 2014 Mar 12;9(3):e90779. doi: 10.1371/journal.pone.0090779 (PMC3951273; doi:10.1371/journal.pone.0090779)
Supplement: File S1 — Supplementary Information, Analysis and Interpretation of PCAs. (DOCX) [file pone.0090779.s001.docx]

# Supplementary Information, Analysis and Interpretation of PCAs.

## How do you say ‘Hello’? Speaker personality impressions from a brief voice sample. McAleer, Belin & Todorov.

For male voices, a three-dimensional solution explained 94.9% of the variance (56.2% by the first principal component (PC1); 31.8% by PC2; 6.9% by PC3) – See Table_S1 (left-panel)). Discussion of PCs 1 & 2 is as in the manuscript. For PC3, Aggressiveness, Confidence, Dominance and Warmth had positive loadings, whilst Attractiveness, Competence, Likeability and Trustworthiness had negative loadings. Using the same method that established Valence and Dominance as summaries of PC1 & PC2, no traits were shown to have a significant correlation with PC3. Furthermore, neither masculinity nor femininity showed a significant correlation to PC3.

For female voices, a three-dimensional solution explained 90.3% of the variance (59.5% by PC1; 25.5% by PC2; 5.2% by PC3) – See Table_S1 (right-panel)). For PC3, Confidence, Likeability and Warmth had negative loadings, whilst all other traits had positive loadings. No rating scale was found to have a significant relationship to PC3. Again, neither masculinity nor femininity showed a significant correlation to PC3.

In summary, the third component cannot be explained using the current traits. In both male and female PCAs, PC3 explained only a small part of the variance, and despite moderate loadings suggesting a relationship to Attractiveness and Confidence, no relationship was found that would summarise a third component. In turn, this would support the acceptance of a two dimensional social voice space for both genders.

## PCA of judgement ratings: Analysis By Gender of Rater

The focus of the manuscript was to examine ratings of personality to male and female speakers, and this was carried out collapsing across the gender of the rater. It is however interesting to examine any changes within rater gender, though lower numbers of male participants compared to female participants, would suggest viewing the results tentatively.

### Participants and Reliability

On average, the number of female to male participants per rating scale was 2 to 1 (see Table_S2). Cronbach Alpha, indicating reliability of judgements across participants within groups, was reasonably high for female raters (all Alphas > 0.75); for male raters, Alpha varied across the medium to high range (all Alphas >0.6).

## Female Voices:

Principal Component Analysis exploring personality ratings of Female voices by Male and Female raters is shown in Table_S3. Analysis was performed as in the main PCAs of the manuscript.

Male raters of Female Voices: a two-dimensional solution was found to suitably summarise ratings for the female voices by male raters, explaining 81.08% of the variance (52.92% by the first principal component (PC1); 28.16% by PC2; 6.37% by PC3). Including PC3 in the solution explained a total variance of 87.45%. All loadings on PC1 were positive except Aggressiveness; on PC2, Aggressiveness, Competence, Confidence, Dominance and Warmth were all positive; on PC3, all ratings loaded positively except Competence, Dominance and Warmth.

For PC1, Trustworthiness (r_s_=.72, p<.01), Likeability (r_s_=.72, p<.01) and Warmth (r_s_=.74, p<.01) had the strongest correlates, though only Trustworthiness and Warmth showed no correlation with either PC2 or PC3. Dominance had the strongest correlation with PC2, and had no correlation with either of the remaining dimensions (Dominance to PC2: r_s_=.85, p<.01). Furthermore, despite a moderate loading of Attractiveness to PC3, no significant correlations were found between any rating scale and PC3. In turn, Attractiveness ratings of female voices by male raters was shown to significantly correlate only with PC1, r_s_=.58, p<.01. Finally, regarding Masculinity and Femininity, PC1 was found to correlate positively with Femininity (r_s_=.71, p<.01) and negatively with Masculinity (r_s_=-.64, p<.01). No correlations were found between Masculinity, Femininity and the remaining two components.

Female raters of Female Voices: a two-dimensional solution was found to suitably summarise ratings for the female voices by female raters, explaining 87.09% of the variance (58.38% by PC1; 28.71% by PC2; 5.06% by PC3 - including PC3 in the solution explained a total variance of 92.15%). All loadings on PC1 were positive except Aggressiveness; on PC2, Aggressiveness, Competence, Confidence, and Dominance were all positive; on PC3, all ratings loaded positively except Confidence, Likeability and Warmth

For PC1, Trustworthiness (r_s_=.87, p<.01), Likeability (r_s_=.90, p<.01), Warmth (r_s_=.83, p<.01) and Competence (r_s_=.81, p<.01) had the strongest positive correlates and all four rating scales showed no significant correlation with either PC2 or PC3. Dominance had the strongest positive correlation with PC2, though also showed a medium positive correlation to PC1 (Dominance to PC2: r_s_=.85, p<.01; Dominance to PC1: r_s_=.53, p<.01). Aggressiveness showed the next strongest positive correlation with PC2, and showed no significant correlation to either PC2 or PC3 (Aggressiveness to PC2: r_s_=.77, p<.05). For PC3, Attractiveness ratings of female voices by female raters showed a weak, but significant, negative correlation (r_s_=-.35, p<.05) though was also found to be positively correlated with PC1 (r_s_=.5, p<.05) and negatively correlated with PC2 (r_s_=-.43, p<.05). No other significant correlations to PC3 were found. Finally, as for male raters of female voices, Masculinity (negatively) and Femininity (positively) were found to hold significant correlation with only PC1 (Femininity to PC1, r_s_=.82, p<.01; Masculinity, r_s_=-.64, p<.01).

## Male Voices:

Principal Component Analysis exploring personality ratings of Male voices by Male and Female raters is shown in Table_S4. Analysis was performed as in the main PCAs of the manuscript.

Male Raters of Male Voices: A two-dimensional solution was found to summarise all ratings for male voices by male raters, explaining 84.46% of the variance (56.45% by PC1; 28.01% by PC2; 7.24% by PC3 – explained variance increases to 91.7% when including PC3). All loadings on PC1 were positive except Aggressiveness; on PC2, all ratings loaded positively except Likeability and Warmth; on PC3, Aggressiveness, Confidence, Dominance and Warmth loaded positively whilst Attractiveness, Competence, Likeability and Trustworthiness loaded negatively.

For PC1, Trustworthiness (r_s_=.83, p<.01) and Likeability (r_s_=.88, p<.01) had the strongest positive correlations and showed no significant correlations with either PC2 or PC3. Dominance again showed the strongest positive correlation with PC2 (r_s_=.86, p<.01) and no significant correlations with the remaining dimensions. No significant correlations were found between ratings scales and PC3. Regarding Attractiveness for male raters of male voices, it was found to have a significant positive relationship with PC1 (r_s_=.65, p<.01) and a weaker, but significant, positive relationship with PC2 (r_s_=.38, p<.05). Finally, Masculinity was found to be negatively correlated with PC1 (r_s_=-.44, p<.05) and positively correlated with PC2 (r_s_=.54, p<.01); conversely, Femininity was found to positively correlate with PC1 (r_s_=.52, p<.05) and negatively correlate with PC2 (r_s_=-.47, p<.05).

Female Raters: A two-dimensional solution was found to summarise all ratings for male voices by female raters, explaining 87.12% of the variance (54.21% by PC1; 32.91% by PC2; 6.86% by PC3 – explained variance increases to 93.98% when including PC3). All loadings on PC1 were positive except Aggressiveness; on PC2, all rating scales loaded positively except for Likeability, Trustworthiness and Warmth; for PC3, Aggressiveness, Confidence, Dominance and Warmth loaded positively whilst Attractiveness, Competence, Likeability and Trustworthiness loaded negatively.

For PC1, Trustworthiness (r_s_=.8, p<.01) and Likeability (r_s_=.87, p<.01) had the strongest positive correlations, though Likeability had a significant negative relationship with PC2 (r_s_=-.38, p<.05). Dominance had the strongest positive relationship with PC2 (r_s_=.89, p<.05) and was not correlated to either PC2 or PC3. No rating scales were significantly correlated to PC3. Looking at Attractiveness in female raters of male voices, only a significant correlation was found with PC2 (r_s_=.64, p<.05). Finally, PC1 was found to negatively correlate with Masculinity (r_s_=-.4, p<.05) and positively correlate with Femininity (r_s_=.58, p<.05); PC2 was found to significantly correlate only with Masculinity (r_s_=.58, p<.05).

## Summary

It would appear that reliability of raters, when split by gender, is at an acceptable but could have been improved with increased participant numbers; in particular male participants. In regards to PCA solutions explaining a social voice space, irrespective of gender of rater or gender of speaker, all PCAs could be summarised by a two dimensional solution that would have a first dimension relating to valence or friendliness, and a second dimension relating mainly to dominance or strength.

Small numerical changes exist between the above PCAs when considering Masculinity and Femininity. However, a suitable synopsis would be that for female voices, an increase in PC1 results in increased femininity and decreased Masculinity; for male voices, Masculinity increases with PC2 and decreases with PC1, with the reverse pattern existing for Femininity. These findings are highly consistent with the PCAs of the main manuscript where gender of rater has been collapsed.

One noticeable difference in the above analyses, between genders, concerns Attractiveness. When rating female voices, both male and female participants show an increase of ratings of Attractiveness along with an increase in PC1 – trust, friendliness. However, when rating male voices, female raters only show an increase in attractiveness ratings to an increase in PC2 – dominance; male raters show an increase in attractiveness ratings to an increase in both PC1 and PC2, with the relationship being strongest between PC1 and attractiveness. This suggests female raters associate male vocal attractiveness with perceived dominance, whilst male raters associate male vocal attractiveness with an increase in perceived trustworthiness and dominance. It must be remembered however, that the number of male participants was small in this sample, and other unaccounted for factors, such as sexuality, may have influence [[1](#_ENREF_1)].

Reference

1. Little AC, Jones BC, DeBruine LM (2011) Facial attractiveness: evolutionary based research. Philos T R Soc B 366: 1638-1659.
