# Supplementary material for: How Do You Say ‘Hello’? Personality Impressions from Brief Novel Voices
Source: PLoS One. 2014 Mar 12;9(3):e90779. doi: 10.1371/journal.pone.0090779 (PMC3951273; doi:10.1371/journal.pone.0090779)
Supplement: Table S1 — A three dimensional solution to the ‘social voice’ space. (DOCX) [file pone.0090779.s002.docx]

# Table_S1: A three dimensional solution to the ‘social voice’ space. Loadings on the first three principal components of eight social traits for the male and female voice PCAs, including variance explained. Loadings represent the correlations of the trait judgements with the first three principal components as calculated including eight personality traits, excluding masculinity and femininity.

|  | Male PCA | | | Female PCA | | |
| --- | --- | --- | --- | --- | --- | --- |
| Social Trait | Component  1 | Component 2 | Component 3 | Component  1 | Component 2 | Component 3 |
| Aggressiveness | -0.74 | 0.61 | 0.07 | -0.52 | 0.76 | 0.11 |
| Attractiveness | 0.33 | 0.71 | -0.4 | 0.74 | -0.45 | 0.41 |
| Competence | 0.70 | 0.63 | -0.28 | 0.88 | 0.20 | 0.26 |
| Confidence | 0.75 | 0.44 | 0.45 | 0.62 | 0.74 | -0.11 |
| Dominance | 0.15 | 0.98 | 0.08 | 0.55 | 0.80 | 0.13 |
| Likeability | 0.95 | -0.20 | -0.11 | 0.93 | -0.24 | -0.03 |
| Trustworthiness | 0.92 | -0.05 | -0.3 | 0.96 | -0.15 | 0.06 |
| Warmth | 0.91 | -0.35 | 0.14 | 0.91 | -0.12 | -0.36 |
| Variance Explained (%) | 56.18 | 31.8 | 6.9 | 59.54 | 28.53 | 5.23 |
