# Supplementary material for: How Do You Say ‘Hello’? Personality Impressions from Brief Novel Voices
Source: PLoS One. 2014 Mar 12;9(3):e90779. doi: 10.1371/journal.pone.0090779 (PMC3951273; doi:10.1371/journal.pone.0090779)
Supplement: Table S2 — Proportion of each gender per personality scale. (DOCX) [file pone.0090779.s003.docx]

**Table_S2: Proportion of each gender per personality scale. Cronbach alpha scores, indicating reliability of judgments, and number of participants per gender per trait judgment. Alpha greater than 0.85 is considered to be high.**

| Social Trait | Female Raters | | Male Raters | | Total |
| --- | --- | --- | --- | --- | --- |
|  | n | alpha | n | alpha |  |
| Aggressiveness | 18 | 0.87 | 15 | 0.82 | 33 |
| Attractiveness | 21 | 0.79 | 10 | 0.7 | 31 |
| Competence | 21 | 0.84 | 15 | 0.79 | 36 |
| Confidence | 22 | 0.89 | 12 | 0.84 | 34 |
| Dominance | 15 | 0.89 | 13 | 0.86 | 28 |
| Femininity | 16 | 0.81 | 8 | 0.78 | 24 |
| Likeability | 21 | 0.77 | 9 | 0.62 | 30 |
| Masculinity | 16 | 0.77 | 9 | 0.67 | 25 |
| Trustworthiness | 17 | 0.77 | 11 | 0.65 | 28 |
| Warmth | 23 | 0.91 | 10 | 0.85 | 33 |
| Average | 19.1 | 0.83 | 11.2 | 0.75 | 30.2 |
