# Supplementary material for: How Do You Say ‘Hello’? Personality Impressions from Brief Novel Voices
Source: PLoS One. 2014 Mar 12;9(3):e90779. doi: 10.1371/journal.pone.0090779 (PMC3951273; doi:10.1371/journal.pone.0090779)
Supplement: Table S3 — A three dimensional solution for female voices by rater gender. (DOCX) [file pone.0090779.s004.docx]

**Table_S3. A three dimensional solution for female voices by rater gender. Loadings on the first three principal components of eight social traits for female voices PCAs as rated by male and female raters, including variance explained. Loadings represent the correlations of the trait judgements with the first three principal components as calculated including eight personality traits, excluding masculinity and femininity.**

|  | Male raters | | | Female raters | | |
| --- | --- | --- | --- | --- | --- | --- |
| Social Trait | Component  1 | Component 2 | Component 3 | Component  1 | Component 2 | Component 3 |
| Aggressiveness | -0.44 | 0.64 | 0.24 | -0.33 | 0.85 | 0.15 |
| Attractiveness | 0.68 | -0.15 | 0.52 | 0.67 | -0.55 | 0.41 |
| Competence | 0.81 | 0.39 | -0.12 | 0.85 | 0.07 | 0.37 |
| Confidence | 0.60 | 0.76 | 0.06 | 0.68 | 0.68 | -0.07 |
| Dominance | 0.40 | 0.89 | -0.14 | 0.67 | 0.70 | 0.06 |
| Likeability | 0.83 | -0.24 | 0.076 | 0.91 | -0.24 | -0.08 |
| Trustworthiness | 0.81 | -0.26 | 0.075 | 0.94 | -0.24 | 0.08 |
| Warmth | 0.86 | 0.05 | -0.34 | 0.89 | -0.16 | -0.36 |
| Variance Explained (%) | 52.92 | 28.16 | 6.37 | 58.38 | 28.71 | 5.06 |
